# Temperament Predicts the Quality of Social Interactions in Captive Female Rhesus Macaques (*Macaca mulatta*)

**DOI:** 10.3390/ani11082452

**Published:** 2021-08-20

**Authors:** Ori Pomerantz, John P. Capitanio

**Affiliations:** 1 California National Primate Research Center, University of California, Davis, One Shields Ave, Davis, CA 95616, USA; jpcapitanio@ucdavis.edu; 2 Department of Psychology, University of California, Davis, One Shields Avenue, Davis, CA 95616, USA

**Keywords:** temperament, sociality, welfare, rhesus macaque, behavioral compatibility, interaction quality

## Abstract

**Simple Summary:**

Rhesus macaques are highly social animals that are used extensively in research. Providing them with a supportive social environment by pairing individuals with compatible partners improves their life quality and also their use as research animals. Therefore, identifying suitable social partners will benefit both the animals and the science. Previous work showed that female rhesus macaques were more likely to form successful pairs that exhibit little aggression if they showed similarities in certain personality traits measured in infancy. In the current study, we assessed the relationships between the same personality traits and the quality of social interactions between partners. This is important since by ensuring that animals benefit the most from being paired to a compatible partner, we are more likely to expose the animals to positive experiences instead of simply avoiding negative ones. We found that pairs with similar emotionality scores showed more positive social interactions, and pairs with similar nervous temperament used fewer behavioral signals to communicate their dominance relationship. Moreover, pairs that paid more attention to unfamiliar monkey faces were more anxious. These results highlight the importance of taking the animals’ personalities into consideration when attempting to match social partners and provide them with an environment that fits their needs.

**Abstract:**

Previous reports suggest that female macaques with greater similarity in emotionality and nervous temperament, as evaluated in a well-established BioBehavioral Assessment (BBA) at the California National Primate Research Center, were more likely to form successful pairs. We tested whether the same measures can also predict the quality of social interactions among 20 female rhesus macaque pairs. We correlated the pairs’ emotionality and nervous temperament scores obtained in infancy and the levels of behaviors recorded systematically during the pairing process years later. Supporting previous findings, partners with similar emotionality scores were more affiliative, and pairs with similar nervous temperament expressed less dominance/submissive behavior. Exploratorily, we found that pairs that were better at processing social information (part of BBA) were also more anxious. Such animals should be prioritized to be introduced in rooms that house calmer, less aggressive animals and provide opportunities for hiding to alleviate their anxiety. Indeed, positive social experiences not only promote animal welfare, but also reduce stress related confounds and unexplained data variability. Therefore, by incorporating the animals’ temperament into the pair configuration process we increase the likelihood of forming high-quality pairs, both in terms of welfare and the research of which they are a part.

## 1. Introduction

### 1.1. Social Buffering and Fitness among Nonhuman-Primates Living in the Wild

Social relationships constitute the backbone of nonhuman primate (NHP) societies. Aspects of sociality such as the number of stable social bonds and their quality have been linked to fitness in several NHP species, including in rhesus macaques (*Macaca mulatta*). It was suggested that by forming social relationships animals have been more successful in coping with selective pressures such as predation; more generally, by utilizing social behaviors NHP can buffer the stress response to the challenges they face in their environment [1]. Indeed, NHP group members that form stable and strong social bonds and are more socially integrated demonstrate higher survival rates and reproductive success in comparison to less social individuals [2] (pp. 127–175). Barbary macaques (*Macaca sylvanus*), for instance, form huddles to shelter themselves from cold and wet weather, and individuals that had more grooming partners were a part of larger, more protective huddles than less social group members [3]. Female baboons (*Papio* spp.) with a high score of relative social connectedness (based on grooming networks) were significantly less likely to die in a given year compared to females with low scores [4]. Similarly, rhesus macaques that frequently groomed and spent time in proximity to others that further groomed others (i.e., friends of friends) had higher reproductive outputs [5]. These fitness related benefits are made possible by the formation and constant maintenance of social relationships. The link between sociality and fitness is mediated by several pathways, including, for example, communal care, cooperation for gaining access to resources, and development of behavioral skills [6]. It has been postulated that animals are highly motivated to express behaviors that increase fitness [7], and that thwarting their expression can lead to frustration and negatively affect the animal’s welfare [8]. Thus, providing a social environment that fits the animal’s needs and enables the expression of highly motivated social behaviors is desirable.

### 1.2. Social Buffering and Welfare among Nonhuman-Primates Living in the Research Environment

Rhesus and Cynomolgus macaques (*Macaca fascicularis*) are the most commonly used NHP species in research [9]. In 2019, for example, approximately 70,000 NHP were used in research in the US [10], mostly in pharmaceutical research and development, neuroscience, vaccine development and immunology research [9]. At the CNPRC, the majority of the animals are housed outdoors, either in half acre enclosures with approximately 60–190 animals, or in smaller enclosures (i.e., corncribs) that house approximately 7–22 animals. The rest of the animals are housed indoors, mostly in pairs, but also in single housing when appropriate (i.e., with the proper exemption). Indoor housing is used primarily for research related procedures. Data from a 2014 survey show that, in the US, roughly 83% of research rhesus macaques were socially housed, and about 50% of research NHP are socially housed indoors [9]. Captive rhesus macaques that are used in research do not typically face similar pressures as their free ranging counterparts. However, they do experience other environmental stressors that may affect their biological functioning and with which they are required to successfully cope to maintain homeostasis and adequate welfare states (e.g., [11,12]). For example, relocating animals to a new room (a highly frequent husbandry activity) has been associated with activation of the hypothalamic–pituitary–adrenal axis, and the resultant increase in cortisol secretion (reviewed in [13]). Further, female rhesus macaques that were repeatedly stressed during pregnancy produced more reactive offspring, as indicated by higher levels of both ACTH and cortisol in response to common environmental stressors in a research setting [14]. Despite the environmental differences, both wild and captive NHP populations rely heavily on conspecifics to help mitigate the stress response. When pair housed, adult male rhesus macaques exhibited significantly lower levels of abnormal stereotypic behaviors in response to observing other monkeys in the room being restrained for administration of an injectable anesthetic (a common procedure in a research setting) in comparison to when they were singly housed [15]. The authors suggested that being socially housed with a pair mate provided the animals with opportunities to better cope with routine stressors and therefore, increased their welfare levels. The expression of affiliative behaviors such as social contact and allogrooming has been shown to be associated with a reduction in levels of hair cortisol among rhesus macaques, suggesting that the expression of affiliative behaviors was involved in the animals’ coping mechanism [16]. Similarly, juvenile rhesus macaques engaged less in solitary behaviors (e.g., self-grooming) and were less stressed (e.g., less screaming) when relocated to a novel cage together with a social partner compared to when they were alone [17]. Indeed, coping and whether it is successful or not is central to the welfare of the animals since it reflects the animals’ ability to modify their response to an aversive situation [18,19].

### 1.3. Coping with Stressors—Implications for Research

Such physiological and behavioral manifestations of the stress response are of particular interest when considering research animals and the ability to generate reliable and meaningful scientific information. Ignoring the potential effects of environmental stressors in a research setting may introduce unaccounted for variables and can ultimately lead to inaccurate conclusions. For example, it is common to maintain NHP in single housing when studying infectious diseases to prevent cross contamination between animals. However, as sociality plays a fundamental role in NHP lives, perhaps the greatest challenge that laboratory NHP are faced with is being housed alone without the comforting presence of a compatible partner [20]. Single housing of NHP is a putative stressor with both physiological and behavioral implications that may skew the data [21,22]. Pig-tailed macaques (*Macaca nemestrina*) infected with simian immunodeficiency virus (SIV) that were exposed to the common psychosocial stressor of single housing exhibited differential progression of pathogenesis than animals that were socially housed [23]. Thus, following SIV inoculation, singly housed macaques had higher viral loads in both the plasma and the CSF, greater decline in CD4 and CD8 T-cell numbers, as well as in total circulating lymphocytes. Moreover, the data produced by the singly housed animals were more variable than those that were obtained from socially housed subjects [23]. These differences in data output from singly vs. socially housed NHP clearly demonstrate the weight of social housing in the production of high quality and less variable scientific data. Further, rhesus macaques that lived in a stable social environment had better immunity as reflected by a lower expression of inflammation associated genes: *IFNG*, *IL6* and *TNF* compared to when they were a part of an unstable social group [24]. These findings additionally emphasize the importance of factoring in the social housing condition (i.e., single vs. pair) and the stability of the social unit when interpreting results [25,26].

### 1.4. Social Housing and the Quality of Social Interactions

Acknowledging the significance of the social environment for both the welfare of NHP and the validity of the scientific data that were obtained from their use, current guidelines refer to social housing as the default housing condition for captive NHP [27]. Accordingly, the proportion of singly housed NHP indoors across research facilities in the US has decreased substantially from 59% in 2003 [28] to 35% in 2014 [9]. This large decrease in singly housed animals (which corresponds to an increase in social housing) in US research facilities has undoubtfully contributed to the welfare of the NHP research population. However, both the *Guide for the Care and Use of Laboratory Animals* (the *Guide*) [27] and the Animal Welfare Act [29] highlight the need to not only house any two individuals together, but also to ensure that the animals are compatible and the pair is stable. For many, compatibility and stability of social partners is determined by the absence of aggression. For example, successful social introductions of research NHP are often defined as those in which social partners were able to remain co-housed for a predetermined period without any injuries or overt aggression [30]. Indeed, the absence of aggression, fear and depression is frequently used as the main requirement for judging a pair to be successful (e.g., [31]). However, in recent years, animal welfare scientists have promoted the notion that “positive welfare” is more than a mere absence of negative experiences, and in fact, should include opportunities for positive experiences [32,33,34]. Thus, rather than focusing solely on reductions in levels of welfare indices such as aggression, abnormal behavior and/or corticosteroids, it is recommended that behavior managers collect additional information pertaining to the expression of behavioral and physiological parameters associated with positive affect (e.g., brain opioids) [35].

The current study sought to build on previous research conducted by us and others [30,36,37,38] that identified predictors of pair success (i.e., can social partners remain together continuously in a shared space without persistent aggression and wounding). In particular, our recent study [36] demonstrated that, for female pair formations, three measures, taken from the California National Primate Research Center’s (CNPRC) BioBehavioral Assessment (BBA) Program (see Section 2.4) predicted success in pairings. These measures were difference scores for emotionality recorded soon after separation and relocation; emotionality recorded during a human intruder challenge; and nervous temperament. These relationships were all negative; pairs whose members were more similar on these three measures (i.e., had smaller difference scores), were more likely to be successful. As we indicated in [36], whether a pair is judged successful or not will depend on the behavior seen during the social introduction process. We did not have behavioral data for that earlier study, but we do for the animals in the present study.

Our first hypothesis, then, was that these three measures exert their effects on pairing success by contributing to greater affiliative behavior, less aggressive behavior, and/or less anxious behavior during the initial periods of contact between prospective pair mates. Since our earlier paper was published, we identified another measure from the BBA Program that strongly predicts, years later, whether an animal will become high or low in social functioning [39]. This is a measure of face recognition and assesses the extent to which animals can discriminate a familiar from a novel face (see Section 2.4). Monkeys are presented with seven problems, and for each problem they see a photo of an unfamiliar animal, after which that picture is paired with a novel picture. The outcome measure for each problem is the proportion of time that animals viewed the novel picture, divided by the total time the animals viewed either picture. A mean of this novelty preference measure was taken for the seven problems. Overall, rhesus macaques (and humans) prefer to look at the novel stimuli in such a test, and we found that on our particular test, animals that showed the novelty preference at 3–4 months of age demonstrated high social functioning in their living cages at 1–4 years of age, while animals that did not show this preference did not [38]. This test, then, tells us something about social information processing, and we hypothesized that it might be influential in pairing success. We calculated both mean and difference scores for each pair and correlated them with behaviors recorded during social introductions.

Our second hypothesis, then, was an exploratory one. Specifically, we tested the relationship between performance on the face recognition task and the behaviors observed during initial stages of the social introduction.

Overall, we believe that identifying predictors of behavior in a pairing context will facilitate the refinement of the social introduction process and pair maintenance procedures and is therefore likely to increase the animals’ welfare and their value as research animals.

## 2. Materials and Methods

All aspects of management and research use conformed to applicable US federal regulations and the guidelines described in the *Guide for the Care and Use of Laboratory Animals* [27] and the US Department of Agriculture’s Animal Welfare regulations [29], and adhered to the study’s protocol as approved by the University of California, Davis Institutional Animal Care and Use Committee (protocol code 20286, 12/14/2017, replaced by 22033, 12/03/2020).

### 2.1. Subjects and Housing

The study subjects were 40 female rhesus macaques (*Macaca mulatta*) (mean age ± SD 10.7 ± 4.1 years) arranged into 20 pairs that were a part of several unrelated research and breeding protocols. All the animals went through the BBA program as infants (see Section 2.4) between 2001 and 2018. All pairs but one were introduced in 2020; one pair was introduced in 2019. Eighteen of the pairs comprised 2 adult females (i.e., >3.0 years of age), and the remaining two pairs involved one adult female and one juvenile female (both juveniles were approximately 2.7 years of age at the onset of the introduction). All but 5 of the females were raised by their dams in an outdoor social group. Three of the remaining 5 females were raised by a dam in indoor housing until their weaning, one was nursery reared and one had mixed rearing. We included all female–female pairs with the following criteria: (a) both partners had data obtained from CNPRC’s long-standing BioBehavioral Assessment Program (e.g., [40] and see Section 2.4); (b) the introduction process included both a protected contact phase and a full contact phase; and (c) behavioral data were collected systematically on the first day of both the protected contact and full contact phases. The animals were housed indoors in rooms maintained on a 12:12 h light:dark cycle with an ambient temperature between 20 and 22 °C and a relative humidity of 30–70%. Subjects were housed in standard stainless-steel caging, which met or exceeded federal animal welfare regulations. The middle wall of each cage was fitted with one solid sliding panel and one metal grate. Prior to the onset of the social introduction (single housing condition), prospective social partners resided in adjacent cages, separated with a solid panel. The solid panel was then removed to allow partial visual and tactile contact through the metal grate. Next, if animals did not show overt and persistent aggression in protected contact, the grate was removed, and the animals progressed to continuous full contact. Study subjects were provided LabDiet 5047 Jumbo primate biscuits twice per day and water ad libitum. Animals were provided with various environmental enrichment items as well as a daily forage board mix and fresh or dried produce twice per week.

### 2.2. Social Introduction Procedure

Prospective partners were identified and matched based on their age, weight, social history, and known trajectory (e.g., whether the animal being considered for study enrollment, whether the animal going to be shipped to another facility, whether the animal planned to be included in new breeding group formation). In addition, since 2018, in response to the findings reported by Capitanio et al. [36], behavioral management staff began incorporating information collected in the BioBehavioral Assessment (see Section 2.4) into the pair configuration process. Particularly, staff attempted to match partners with similar scores in Day 1 emotionality, nervous temperament, and emotionality recorded during the Human-Intruder test (see Section 2.4), as these have been shown to be strong predictors of pair success [36]. A minimum of 24 h prior to the onset of the introduction process, the prospective partners were placed in adjacent cages. Social introductions between potential pair mates were conducted between Mondays and Thursdays in the mornings (9:00–11:00 AM) after husbandry work was completed in the rooms. Behavioral management staff began introductions by pulling the solid panel, permitting limited contact through the metal grate, thus entering a protected contact phase. While in protected contact, prospective partners could touch, communicate, and interact through the grate. Introduced animals were monitored closely immediately after the onset of the introduction and over several days thereafter. Additional observations were conducted based on the judgment of the behavioral management staff who introduced the animals. In addition to systematic data collection (described in Section 2.3), checks included visual inspection of the animals and the cage for blood and injuries. None of the pairs exhibited obvious traditional signs of incompatibility such as overt, persistent, bi-directional aggression during the protected contact phase. Therefore, all progressed to the continuous full contact phase by removing the grate. This step was taken after a mean of 4.8 ± 3.9 days in protected contact. The duration of this phase was influenced by two factors: (1) the schedule of upcoming anesthetic or husbandry events that might negatively influence introduction outcomes by increasing the stress experienced by the animals; and (2) observed behavior.

### 2.3. Behavioral Data Collection

Data for each animal were collected at two time points: during the first 15 min of the initial protected contact introduction, and during the first 15 min of the initial continuous full contact phase. Each pair was observed by one of three behavioral management technicians, who were trained by the first author of this study to identify behaviors of interest as part of routine behavioral management training (see Table 1). A high level of inter-observer reliability was maintained across all observers for all behavioral data collection (Krippendorf’s alpha ≥ 0.80). The behavioral management technicians entered the room and waited approximately 10 min prior to the onset of data collection which began immediately following the pulling of the solid panel (the onset of protected contact). A total of 1200 min of data were recorded in person on a tablet computer, using one-zero sampling with 60 s intervals [41] (pp. 48–60).

### 2.4. BioBehavioral Assessment

The BioBehavioral Assessment (BBA) Program at CNPRC was established in 2000, with the first year of data collection in 2001; a total of more than 5000 animals have been assessed to date. The goals of the program were first, to obtain data on aspects of biobehavioral organization, a concept that includes intrinsic characteristics of the individual such as coping abilities, stress reactivity, temperament, hypothalamic-pituitary-adrenal integrity, cognition, personality, etc.; second, to make these data available to scientists for use in subject selection and for colony management personnel to use for better management; and third, to explore the causes and consequences of this variation. Details of the program have been published elsewhere (e.g., [40]), including in this Special Issue [42].

Briefly, infants (90–120 days of age) are relocated from their living cages to a standard adult female-size individual cage indoors in cohorts of 5–8 individuals for a period of 25 h. Animals are tested individually (in a pre-determined random order) on a battery of tests that assess aspects of biobehavioral organization. At the end of the testing period, animals are returned to their original living cages. The 40 female monkeys in the present study were evaluated in the BBA Program at a mean age of 105.33 days (SD = 10.1 days, range = 90–120 days). We describe next the four assessments from this program that are pertinent to the present report.

Holding Cage: After the animals have been in their holding cages for approximately 15 min, they are observed for 5 min each using focal animal sampling. Exploratory and confirmatory factor analyses of these data [43] identified two factors, activity and emotionality, that described the behavioral data. These observations are repeated on the second day of the assessment; day 1 measures reflect the animals’ responsiveness to the separation and relocation associated with participation in the BBA Program, and day 2 measures reflect the animals’ adaptation. The emotionality score from this first day of observation (referred to as D1E, that is, day 1 emotionality) was one of the predictors in our earlier study [36], and comprises the behaviors coo, bark, scratch, threat, and lip smack. The relationship between activity related measurements and the quality of social interactions was not evaluated in the current study since activity was not predictive of pairing success as reported in [36].

Face Recognition: Also known as a test of visual recognition memory or a “preferential looking” task, this test is conducted after the holding cage observations. Each monkey sees a pre-recorded video that presents seven problems. For each problem, the subject is presented with identical pictures side-by-side on a monitor for 20 s, after which the screen goes blank. Next are two 8 s test trials, in which the now-familiar picture and a novel picture are presented; the two trials differ only in the placement of the stimuli (left vs. right side of display). The typical response of young monkeys (and humans) is to spend more time looking at the novel picture in the test trials, and the outcome measure is the proportion of total looking time that animals look at the novel stimuli (i.e., duration of looking at novel divided by duration of looking at novel + familiar stimuli in the test trials). The outcome measure is the mean of this looking response across the seven problems and is referred to as NP (novel preference), reflecting the preference for the novel stimuli (we note that because our animals are unrestrained for this test, they could choose to look at the stimuli or not; we only included their responses to a problem if they showed any duration of looking at either the left or the right image on the first 20 s trial for any problem). This is a test of face recognition because our stimuli are photos of unfamiliar conspecifics with neutral expressions.

Human Intruder. This social challenge test occurs mid-afternoon during BBA testing and comprises four one-minute trials. A technician presents her face in profile for one minute from ~1 m from the monkey, after which she moves closer (~1/3–1/2 m), in the same orientation, for a second minute. Then, returning to the far position, she stares at the animal for one minute before moving to the near position for the fourth minute. Exploratory and confirmatory factor analyses of these data [44] revealed four factors, one of which was emotionality (abbreviated at HIE), comprising the behaviors coo, self-clasp, convulsive jerk, and fear grimace.

Temperament. At the end of the 25 h assessment, the technician rates each animal on a 1–7 scale for each of 16 behavioral traits (5–8 animals at a time) designed to assess temperament [43] (Supplementary File 1). Exploratory and confirmatory factor analyses, reported in [43], revealed four dimensions, one of which is nervous temperament (abbreviated NERV), which is composed of high ratings for nervous, fearful, timid, and low ratings for calm and confident. These ratings are done out of sight of the animals, and the technician is instructed to utilize her total experience with the animal: not only how the animals behaved during the various assessments, but also how they responded during feeding, hand-catching (both for phlebotomy and temporary relocation to a test cage for some of the other assessments), blood sampling, and when other animals were being handled for the same procedures.

### 2.5. Statistical Analysis

We conducted a correlational analysis between the BBA measures and the behavioral measures recorded during the pairing process. For the four BBA measures, a difference score was computed for each pair by taking the absolute value of the difference for each pair-mate, resulting in D1Ediff, NPdiff, HIEdiff, and NERVdiff. In addition, we calculated a mean score for NP (NPmean) for each pair. For the behavioral measures, mean values were computed for each pair for the five behavioral categories described in Table 1 for the protected contact and for the continuous full contact conditions. A preliminary correlational analysis, however, revealed similar patterns of correlations between our predictor variables and the behaviors in the protected contact and full contact conditions, consequently, to reduce type I error, we averaged the five behavior categories across the two conditions.

The analysis involved computing Pearson product-moment correlations between BBA measures and behavior measures. Several measures were not normally distributed, however, as indicated by Shapiro–Wilks tests. This included D1Ediff and HIEdiff among the BBA measures, for which the majority of animals had very low values. Log transformation improved the distribution for D1Ediff, but HIEdiff could not be improved. For the behavioral measures, the aggression, dominance, and abnormal measures each had large numbers of zeros, which transformations cannot help. Consequently, for analyses involving HIEdiff and the aggression, dominance, and abnormal behavior measures, a nonparametric correlation (Spearman’s rho) was computed.

Our first analysis focused on D1Ediff, HIEdiff, and NERVdiff, as described earlier. We had specific directional hypotheses and predicted that smaller difference scores would be associated with lower aggression, lower anxiety, and greater affiliation during the pairing attempts. These predictions were based on our earlier study [36] and the statistical tests were one-tailed. For the other outcome measures (abnormal behavior and dominance/submissive behavior), tests were two-tailed. Our second hypothesis was that the two NP-associated measures (NPdiff and NPmean) were related to behavior during pairings and were two-tailed. Alpha was set to 0.05.

## 3. Results

### 3.1. D1Ediff, HIEdiff, and NERVdiff with Behavioral Measures

**Figure 1:**
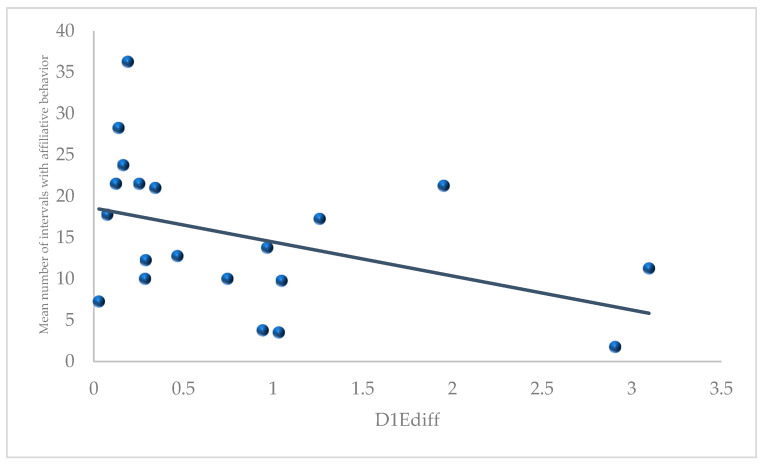
Correlation between difference in day 1 emotionality (D1Ediff) scores between social partners, measured in infancy and their mean number of intervals with observed affiliative behavior averaged across the protected and continuous full contact stages, measured in sub/adulthood (data are presented in the raw form).

Our first hypothesis was that the three measures found to predict successful parings in our earlier study [36] would be associated with more affiliation, and less aggression and anxiety. This hypothesis received limited support in this analysis: pairs whose members had more similar scores (i.e., a smaller difference score) on the day 1 emotionality measures (D1Ediff) displayed higher levels of affiliative behaviors (Pearson r = −0.420, *p* = 0.033, 1-tailed; see Figure 1 and Supplementary Table S1).

**Figure 2:**
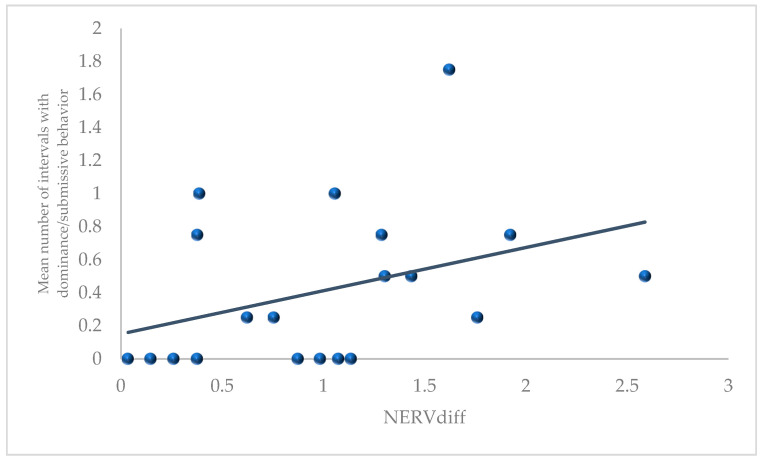
Correlation between difference in nervous temperament scores (NERVdiff) between social partners, measured in infancy and their mean number of intervals with observed dominance/submissive behavior averaged across the protected and continuous full contact stages, measured in sub/adulthood. (Please note that the analysis was done on ranked data; however, the data presented here are raw).

In addition, we found that the more discrepant that pair mates were on nervous temperament (NERVdiff) (i.e., the larger the difference score), the more dominance/submissive behaviors were displayed (Spearman rho = 0.458, *p* = 0.042, 2-tailed; Figure 2 and Supplementary Table S1). No other correlations were significant.

### 3.2. NPmean and NPdiff with Behavioral Measures

**Figure 3:**
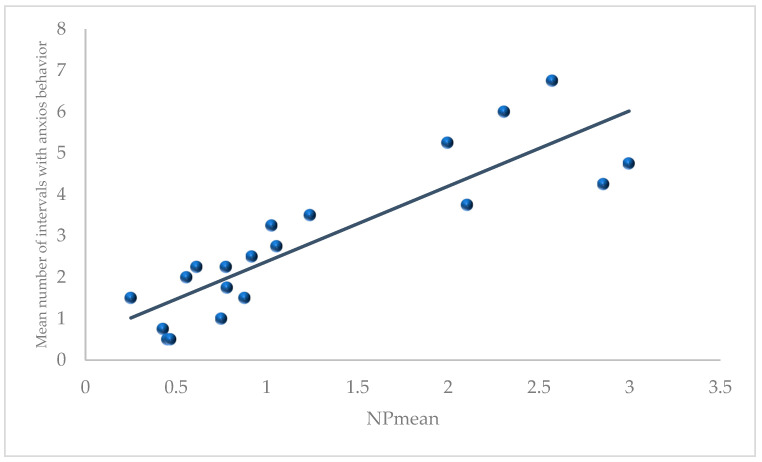
Correlation between mean scores for NP for each pair and the mean scores for anxious behavior averaged across the protected and continuous full contact stages.

Our second hypothesis was exploratory and aimed at determining whether another measure from the BBA Program, NP from the face recognition assessment, was associated with behavior during pair formation. We found that the pair’s mean value on this measure (NPmean) was significantly correlated with frequencies of anxious behavior (Pearson r = 0.503, *p* = 0.024, 2-tailed; Figure 3 and Supplementary Table S1); pairs that were more proficient at detecting novel faces displayed more anxious behavior during pair formation. No other correlations were significant.

## 4. Discussion

### 4.1. Using Levels of Behavior as the Response Variable

The current study provides support for previous research that demonstrated that female rhesus macaques with similar temperament were more likely to form successful pairs [36]. However, rather than using success as the outcome variable, we examined how temperament affects the behavioral output exhibited during the introduction process of prospective social partners. We elected to focus on behaviors that were expressed during the introduction process rather than introduction outcome (i.e., successful vs. unsuccessful) since we were interested in objectively and explicitly assessing the link between various aspects of the animals’ temperament measured during infancy and the quality of their social interactions, as observed years later. By doing so, we were able to assess whether pairing female rhesus macaques provides opportunities for positive experiences (and consequently, increase positive welfare) in addition to a reduction in negative ones. Studies that used introduction outcome as the response variable concentrated on whether pair-mates could remain together for a certain period following the termination of the introduction process (e.g., [36,37]). The determination of whether partners can remain co-housed is made according to the judgment of the behavioral management technician who introduces the animals. While such decisions are certainly influenced by the behaviors observed during the monitoring of introduced animals, some variance in evaluation and interpretation likely exists between technicians. In addition, emphasis is often put on levels of observed aggression (or lack thereof) when judging the outcome of the social introduction. Accordingly, so long as no overt and persistent aggression is observed, social partners tend to be left together even in the absence of affiliative interactions between them [45]. Moreover, the criterion for success, as it has been used, is somewhat arbitrary and ranges from the ability of partners to stay together in a shared space for a few days [36] to 4 weeks [37], and even months [46]. Therefore, methodical collection of behavioral data at different stages of the introduction process (as well as later, when the pairs have been together for some time) will enable behavioral managers to assess the quality of social interactions on a measurable scale independent of human interpretation. Previously, it was reported that similar associations between temperament and behavioral/social compatibility also occur among animals living outdoors in large social groups (i.e., higher similarity in temperament was associated with higher levels of affiliation [47]). However, we stress that the relatively small sample size used in this study warrants caution in generalizing the current findings to more complex social environments than indoor pair housing.

### 4.2. Day 1 Emotionality and Affiliative Behavior

We found that pairs with relatively similar responsiveness scores for day 1 emotionality expressed more affiliative behavior than pairs with more distinct scores. Day 1 emotionality scores were composed of rates of cooing and barking, as well as whether or not the animal scratched, displayed threats, and lip smacked as they were found to be related to each other using factor analyses [43]. Emotionality scores were obtained from observations on the first day of exposure to the novel environments as part of the animal’s BBA (in infancy). The scores were then used as an estimate of the animal’s sensitivity and initial responsiveness to the separation from the dam or social partners and relocation to a novel cage (e.g., [43]). Our result is in line with previous findings that revealed parallel relationships between closeness in such scores and the ability of social partners to remain together in a shared space for a predetermined period. Indeed, female rhesus macaques that scored similarly on day 1 emotionality and nervous temperament were more likely to be deemed a successful pair [36]. Correspondingly, at another facility, rhesus macaques with similar “behavioral inhibition scores” assessed via the animals’ reaction to a novel object were also more likely to remain together (i.e., successful pairs) [46], and rhesus macaque yearlings exhibited preference to engage in affiliative behaviors with peers with similar degrees of equability and adaptability [47]. Together, these results strongly suggest that early characterization of the animals’ responsiveness and temperament is an important step in the configuration of prospected social partners.

It has been suggested that resemblance in temperament between individuals may be at the heart of animal sociality [48]. A potential explanation for this link is that similarity in temperament traits may serve as an indication of the partner’s quality [48]. Moreover, capuchin monkeys (*Sapajus* sp.) with comparable scores of “openness” (a personality dimension) (as well as age and social rank) were found to be more compatible than dyads with dissimilar scores. The authors suggested that these animals may have expressed less agonism and higher levels of relationship quality due to shared interests in each other’s activities [48]. In addition, animals with similar temperaments may be more proficient in predicting each other’s behavior than those that are less similar since both are likely to react comparably to various stimuli. Indeed, animals’ preference for predictable environments and their positive effects on welfare have been widely acknowledged [49]. Therefore, incorporating information about temperament and responsiveness into the social introduction process is thus likely to yield pairs of monkeys that can not only remain together, but also engage in positive prosocial behaviors.

### 4.3. Affiliative Behavior, Coping and Welfare

Traditionally, animal welfare scientists have explored ways of reducing the frequency and magnitude of aversive experiences and then measuring the impact of said reduction on the animals by employing indicators of poor welfare (e.g., abnormal, and anxious behaviors [50,51]). In recent years, however, researchers have been advocating for providing captive animals with opportunities to experience positive states in addition to reduction in negative ones in order to have adequate welfare [34,52,53]. For example, by providing NHP with opportunities to express affiliative behaviors such as allogrooming, animals were able to form and maintain social relationships, which aided in coping with various challenges [54]. Similarly, the authors of the current article often observe animals hide behind social partners, embrace each other, groom and co-threaten when confronted with a stressor as part of their coping (personal communication). Furthermore, affiliative behaviors are regarded as pleasurable and promoting biological functioning [55]. Accordingly, free ranging female chacma baboons (*Papio ursinus*) that experienced social instability due to changes in the alpha male’s social rank were able to alleviate the associated stress response by engaging in affiliative behavior with preferred partners [56]. Therefore, the results of the current study suggest that by pairing social partners with similar day 1 emotionality scores, we were able to offer them a suitable platform to engage in behavioral mechanisms known to be rewarding, facilitate successful coping, and thus promoting welfare (potentially mediated by the release of opioids [35,57]). Hence, it is not surprising that paired rhesus macaques prefer to be in close proximity to their social partners despite the associated cost (in this case, a reduction in space as both animals are in the same half of the pair-cage) [58]. Moreover, a 10% increase in the expression of affiliative behaviors among female rhesus macaques was associated with approximately 3% decline in urinary cortisol levels, further supporting the notion that affiliative behaviors are associated with a positive experience [45].

It should be noted that since BBA information was considered at the time pairs were configured, the ranges of the values for D1Ediff, HIEdiff, and NERVdiff were quite narrow (i.e., in comparison to [36]). The range for D1Ediff in [36], which had 169 female pairs, was approximately 35% higher than in the current sample. Parallel numbers for NERV and HIE in [36] are 75% and 51%. Thus, for D1Ediff, half of the pairs had difference scores of 0.34 or less (maximum difference was 3.1), for HIEdiff, half of the pairs had difference scores of 0.14 or less (maximum difference was 4.14), and for NERVdiff, half of the pairs had difference scores of 0.98 or less (maximum difference was 2.59). These restricted ranges reflect the incorporation of BBA data into the pair configuration process. We suspect that the narrower range of the scores in the present study, owing to this being the very information that technicians used to select prospective pairs (i.e., the narrower range in BBA data among the study animals was not random, but rather stemmed from a deliberate pair configuration based on similar BBA data), may be the reason for the lack of significant correlation between HIEdiff and the levels of observed behaviors during the social introduction.

### 4.4. NERVdiff and Dominance/Submissive Behaviors

The current analysis revealed that pairs with a larger difference score in nervous temperament displayed more dominance/submissive behaviors than pairs with smaller difference. Rhesus macaques are characterized by having a linear dominance hierarchy [59] that is continuously maintained by the expression of behaviors that signal the animal’s social rank in relation to other group members. Beisner and McCowan [60] reported that in social groups of captive rhesus macaques, animals employ silent bared-teeth display (also known as fear-grimacing) to communicate subordination and submission (depending on the context). Similarly, Pomerantz and Baker [38] found that higher levels of submissive behaviors expressed at the early stages of the introduction process were associated with reduced likelihood of altercations (resulting in wounding) between rhesus macaque social partners. We emphasize that the pairs in the current study did exhibit such behaviors during the introduction. However, we note that those pairs with greater similarity in nervous temperament, perhaps required lower levels of expression to establish their dominance relationship. Our finding is therefore harmonious with the concept that similarities in temperament contribute to social compatibility among pairs of rhesus macaques. It could be that social partners with similar nervous temperament scores exhibited more subtle behaviors to signal their relative social status. Alternatively, one can argue that having similar nervous scores reduces the likelihood of social conflict and thus obviates the need to signal their dominance relationship using displays and/or silent bared teeth displays. Further research is required to test if similarities in nervous temperament scores enables social partners to communicate their dominance relationship using different behaviors and at lower intensities.

### 4.5. Social Information Processing and Anxious Behavior

Our results indicate that pairs with relatively higher mean scores for the proportion of time looking at novel faces exhibited higher rates of anxious behavior during the introduction process. Previously, it was reported that rhesus macaques that expressed relatively high levels of pro-social behavior spent more time looking, years earlier, at the novel stimulus in comparison to individuals that exhibited lower levels of pro-social behaviors [39]. The authors suggested that highly social individuals can thus process salient information in their environment more adequately by directing more attention to novel stimuli [39]. Attending to novel (and potentially threatening) stimuli is an adaptive response, which enables the animal to respond appropriately and rapidly [61]. Sclafani et al. [39] demonstrated that infant monkeys that were classified as high social were also more vigilant for threatening social cues, and others have shown that heightened vigilance for threatening stimuli induces anxiety [62]. Indeed, it is common for animals that live in the same room in which the introduction takes place to direct aggression in the form of threats toward the newly formed pair. Thus, it is conceivable that those animals that were found to be more attentive to novel social stimuli as infants maintained this trait through adulthood, and as a result, had increased exposure to threatening social stimuli, which led to an increase in anxious behaviors. We recognize, of course, that this was an exploratory analysis on a relatively small number of pairs and requires replication. Considering this finding, behavioral management staff can identify those individuals with higher affinity for looking at novel faces and modify the social introduction environment in a manner that supports their needs. In this case, the use of visual barriers such as privacy panels is likely to reduce the degree of anxiety that results from threats of neighboring conspecifics [63,64]. In addition, one may want to choose a location where the neighboring animals are of a calmer nature with a lesser tendency for reactivity. By incorporating these aspects into the introduction process, staff is more likely to improve the welfare of the animals, and potentially strengthen the bond between the introduced social partners [63].

### 4.6. Implications for Research

Many publications have demonstrated the crucial role that sociality plays in NHP lives [65,66,67]. Accordingly, ample evidence exists regarding the impact of the degree and type of social interactions on behavioral and physiological indices [14,15,17,21,22,23,68,69]. Clearly, ignoring such effects will cloud the outcomes of studies, as it will inevitably introduce unaccounted for confounds into the data analysis [70,71]. The current study provides additional support to this body of literature by showing the manner in which specific configurations of prospective social partners can affect behavioral output. While not measured here, it is likely that rhesus macaques that exhibited variable degrees of affiliative and anxious behaviors, also differed in physiological output. For example, Debray et al. [72] found that female rhesus macaques that spent more time in social grooming had higher copy numbers of mitochondrial DNA in B cells, demonstrating the effects behavior has on physiological functioning. Therefore, it is reasonable to expect that with a more detailed characterization of the temperament of NHP in the laboratory (in addition to factors such as rearing, current housing condition, and the existence of abnormal behavior), researchers will be able to produce more valid and reliable results that can later be reproduced by others. Refined restraint procedures as well as increases in positive human–animal interactions among long-tailed macaques (*Macaca fascicularis*) that were used in toxicology research yielded less inter-individual variability in the measured outcome, and therefore produced more reliable and precise results [73]. Likewise, Grandi and Ishida [74] reported that positive human–animal interactions in the form of grooming resulted in lower heart rates and higher heart rate variability in two male rhesus macaques. We do note, however, that more studies involving NHP are required to assess the influence of intrinsic (e.g., temperament) and extrinsic (e.g., social environment) factors on measured variables in research to reach a “critical mass” of input that can later be used to make programmatic modifications.

Finally, the importance of using NHP in biomedical research has been firmly established [75], and this issue has become even more pronounced with relation to the recent COVID-19 pandemic that revealed a debilitating shortage of NHP required for biomedical research [76]. One potential way to meet the demands is lowering the number of animals that are needed to achieve an appropriate sample size. This can be done by in-depth characterization of study animals, which will reduce data variability, and enroll animals with particular qualities to studies that fit those qualities. Reduction in number of animals is one of the 3Rs principles, and is, therefore, also expected to benefit their welfare [42,77].

### 4.7. Implications for Welfare

In the current study, we demonstrated that the incorporation of BBA information into the pair configuration process (i.e., the process in which prospective social partners are identified based on a variety of factors such as weight, age, research protocol constraints) increases the likelihood to form successful pairs that benefit from positive experiences. NHP that engage in positive social interactions are likely to have superior welfare over pairs of monkeys that simply “tolerate” each other (although the latter is, in most cases, more beneficial than single housing). By considering the animals’ day 1 emotionality and nervous temperament obtained via BBA, we were able to form pairs of female rhesus macaques that engaged in high levels of affiliation and low levels of dominance/submissive behaviors. The expression of affiliative behavior in NHP has been associated with increased welfare levels and better coping through social support. For instance, juvenile squirrel monkeys (*Saimiri sciureus*) separated from their peers and placed in a novel environment with a social partner produced less “isolation peeps”, a form of distress call, compared to monkeys that were separated and housed alone in a novel environment [78], supporting the stress buffering effect of prosocial contact [34]. Indeed, social grooming leads to a reduction in heart rate and in anxious behaviors [79]. Conversely, the expression of dominance/submissive behaviors such as display, have been associated with an increase in social tension in several NHP species [80]. Therefore, early identification of prospective social partners will enable the formation of pairs that can utilize each other’s company to cope with aversive stimuli and engage in positive experiences. Further, we found that pairs of animals that spent more time looking at novel faces were also more anxious. *A priori* knowledge of such characteristics should lead to proper environmental modifications (e.g., visual barriers, location within a room) aimed at alleviating anxiety associated with unfamiliar neighbors.

Moreover, increasing the likelihood of the formation of successful and compatible pairs has an additional indirect positive effect on the animals’ welfare by reducing the number of additional social introduction attempts that can be stressful in the initial steps, as well as the location changes that are required to accommodate future pairing attempts.

Finally, using data from assessments such as the BBA can also be used to identify individual NHP with specific set of traits that make them more fit for particular studies [77]. For example, Coleman et al. [81] reported that “inhibited” rhesus macaques (as measured by the latency to inspect a novel object) were less likely to learn a simple task with positive reinforcement training techniques than more “exploratory” ones.

## 5. Conclusions

In summation, the current study provides additional support to previous reports that demonstrated that similarities in intrinsic characteristics like temperament predict behavioral compatibility in NHP. Since behavioral compatibility among social partners influences both the animal’s ability to successfully cope with challenges associated with the research environment and with the animal’s biological functioning, researchers are encouraged to incorporate measures of such characteristics into the study’s inclusion/exclusion criteria to increase the welfare of the animals and the quality of data.

## Figures and Tables

**Table 1:** Abnormal, aggressive, anxious, dominance/submissive, and affiliative behaviors recorded during social introductions.

Abnormal Behavior	
Collapses self-hair plucking, self-injurious behavior, repetitive abnormal behavior, and any abnormal idiosyncrasies.	
**Aggressive Behavior**	
Aggressive contact/biting	Physical contact involving biting or biting attempts. Includes “mouth fight” in protected contact.
Aggressive contact/no biting	Physical contact without involvement of the mouth (e.g., pushing, pulling, grabbing, and scratching).
Threatening	At least one of the following partner-directed gestures: ears flattened against the head, brow retracted, open-mouth stare, head bobbing, slap surface or slap at the partner without making contact, and lunging (high-speed aggressive intention movement toward another animal).
**Anxious Behavior**	
Body shuddering	A quick shake of the body.
Scratching	Vigorous back-and-forth movement of nails on one’s own body.
Yawning	Monkey opens mouth wide, often exposing teeth. The head is usually tossed back, and this is sometimes accompanied by a flexing of the open mouth.
Teeth grinding	Clenching teeth with noise from moving jaw.
Dominance/Submissive behavior	
Displaying	Vigorous shaking, slamming, or bouncing off the cage.
Fear grimacing/Silent bared teeth	Grin-like facial expression involving retraction of the lips, exposing teeth.
**Affiliative Behavior**	
Co-threatening/solicit co-threat	Alternating threats and glancing at the partner, that may or may not join in the threatening.
Grooming	Manipulating, brushing, or licking of fur (or eyes, wounds) of another animal with the mouth and/or both hands. Includes both groomer and animal receiving grooming.
Lip-smacking	Bringing the lips together rapidly, resulting in a smacking sound; teeth are covered. Directed at potential partner.
Mounting	With or without pelvic thrusting and with or without foot clasp. Includes both mounter and animal being mounted.
Playing/play soliciting	Non-aggressive, lively actions performed with another individual with or without direct physical contact (e.g., chasing), without pilo-erection, but with relaxed facial expressions.
Rump presenting	A posture involving a stance on all fours with the hind quarters elevated and the tail raised. In some animals the tail may be lifted to the side rather than raised. In some instances, animals may place their heads between their legs.
Proximity	Sitting or lying in the same cage on the same level (both on floor of cage or both on perch) and not engaging in aggressive interaction (i.e., threatening or aggressive contact) for at least 5 s.
Touch Grate	Animal touching grate, excluding hands, hands in a relaxed position.

## Data Availability

The data presented in this study are available on request from the corresponding author.

## Supplementary Materials

The following are available online at https://www.mdpi.com/article/10.3390/ani11082452/s1, File S1: Temperament Ratings; Table S1: Biobehavioral assessment data collected in infancy (Z scores) and behavioral data recorded during the social introduction process (protected and full contact) among the 20 female pairs included in the study (number of intervals in which the behavior was recorded).
