# Supplementary material for: Temperament Predicts the Quality of Social Interactions in Captive Female Rhesus Macaques (Macaca mulatta)
Source: Animals (Basel). 2021 Aug 20;11(8):2452. doi: 10.3390/ani11082452 (PMC8388696; doi:10.3390/ani11082452)
Supplement: Supplementary file 1 [file animals-11-02452-s001.zip › animals-1331098-supplementary.pdf]

Supplementary File S1 - Temperament Ratings

At the end of the 25-hr assessment, each animal was rated on a 1-7 scale for each of 16 traits.

Active, energetic: Moves about a lot, distance traveled by walking, running, climbing, or jumping. Not lethargic.
Aggressive: High frequency of displays, threats.
Bold: fearless; Is daring, not restrained or tentative. Not timid, shy, or coy.
Calm, equable: Reacts in an even, calm way; is not easily disturbed. Not agitated. Restful, peaceful.
Confident: Behaves in a positive, assured manner, not restrained or tentative.
Curious, exploratory, inquisitive: Readily explores new situations, seeking out or investigating novel situation.
Depressed: Subject appears isolated, withdrawn, sullen, brooding, and has reduced activity. Fearful: Fear grins; retreats readily from others or from outside disturbances.
Flexible, not rigid: Adapts to situations. Is able to accommodate new ways of doing things.
Gentle: Subject responds to technicians in an easy-going, kind, and considerate manner. Subject is not rough or threatening.
Nervous, anxious, not calm: Jittery, anxious, seems to be anxious about everything.
Playful: Engages in self-play (hanging, swinging, jumping), or object play.
Slow: Moves and sits in a relaxed manner; moves slowly and deliberately, not easily hurried.
Tense: Shows restraint in posture and movement; carries the body stiffly, which suggests a shrinking tendency, as if trying to pull back and be less conspicuous.
Timid: Subject is easily alarmed and is hesitant to venture into new situations.
Vigilant, alert: Ready, attentive, watchful, notices with special attention. Not oblivious to surroundings.

Supplementary Table S1 - Biobehavioral assessment data collected in infancy (Z scores) and behavioral data recorded during the social introduction process (protected and full contact) among the 20 female pairs included in the study (number of intervals in which the behavior was recorded). Each pair is represented in two rows, one for each subject.

				Biobehavioral assessment data				Protected contact phase				Full contact phase			
Pair ID	Animal ID	Partner ID	Date of birth	Day 1 emotionality	Nervous temperament	Human intruder emotionality	Novpa	Affiliative behavior	Anxious behavior	Dominance/subordinate behavior	Abnormal behavior	Affiliative behavior	Anxious behavior	Dominance/subordinate behavior	Abnormal behavior
1	1a	1b	04/22/10	1.18684	2.43758	-0.45227	0.71	10	0	0	0	10	2	0	0
1	1b	1a	03/30/12	0.72097	2.06302	2.95997	0.68	16	7	0	1	15	8	0	0
2	2a	2b	04/04/07	-0.74956	-1.55460	-0.39174	0.56	2	4	0	0	6	1	2	0
2	2b	2a	03/14/13	-0.72048	-0.25220	-0.53651	0.53	7	1	0	0	14	1	0	0
3	3a	3b	04/06/03	-0.36519	0.15104	-0.41347	0.60	22	7	0	0	26	3	0	0
3	3b	3a	03/27/12	1.58568	-0.92253	1.80109	0.66	10	1	0	0	27	0	0	0
4	4a	4b	03/08/07	-0.44464	0.75165	-0.39174	0.75	2	1	0	1	8	2	0	0
4	4b	4a	04/15/08	0.58674	0.60645	-0.50557	0.90	3	3	0	0	1	0	0	0
5	5a	5b	04/08/10	-0.80276	1.11820	-0.45227	0.49	8	7	1	0	12	6	0	0
5	5b	5a	06/30/10	2.29255	2.87599	-0.45227	0.68	9	7	0	0	16	1	0	0
6	6a	6b	07/10/06	-0.22686	-0.55591	-0.09346	0.50	28	1	0	1	39	0	0	0
6	6b	6a	04/04/07	-0.41790	-1.31181	-0.39174	0.58	29	5	1	0	49	0	0	0
7	7a	7b	03/23/10	0.93923	-0.33825	-0.11329	0.55	17	7	0	0	31	1	0	0
7	7b	7a	06/16/17	0.80262	-0.59695	-0.59995	0.61	26	0	0	0	39	1	0	0
8	8a	8b	03/23/04	-0.05922	-0.84528	-0.32587	0.55	14	8	0	0	19	3	0	0
8	8b	8a	03/23/10	-0.22580	0.28902	-0.45227	0.34	27	2	0	0	35	0	0	0
9	9a	9b	06/26/05	0.50073	1.33652	0.42354	0.72	13	2	0	0	20	1	0	0
9	9b	9a	04/27/10	0.15628	0.35572	-0.45227	0.49	24	6	0	0	27	0	0	0
10	10a	10b	03/09/05	0.11610	-0.42141	-0.14369	0.61	14	5	0	0	14	1	0	0
10	10b	10a	05/15/11	1.37665	1.19891	-0.31182	0.67	22	2	7	0	19	0	0	0
11	11a	11b	03/07/10	-0.80276	0.90366	-0.45227	0.47	0	3	0	0	11	1	0	0
11	11b	11a	03/29/11	-0.51857	-1.68364	-0.39780	0.57	16	7	1	1	13	3	1	0

12	12a	12b	05/04/13	-0.77885	0.25949	-0.53651	0.64	24	15	2	0	18	9	2	0
12	12b	12a	03/12/14	-0.65539	0.64767	-0.48387	0.83	24	3	0	0	20	0	0	0
13	13a	13b	04/12/06	-0.83874	0.18708	-0.42721	0.35	4	0	1	0	1	1	0	0
13	13b	13a	05/13/10	2.06684	-0.43370	-0.45227	0.46	2	2	0	0	0	0	0	0
14	14a	14b	04/17/02	-0.18922	1.55485	-0.42905	0.40	16	1	0	0	22	1	0	0
14	14b	14a	04/02/06	-0.26687	0.68138	1.01748	0.34	10	0	0	0	23	0	0	0
15	15a	15b	07/10/10	1.82472	-0.09847	-0.45227	0.44	8	1	0	2	7	1	0	0
15	15b	15a	03/21/13	0.77520	-0.06254	-0.53651	0.54	20	0	0	0	4	0	0	0
16	16a	16b	04/02/08	-0.48695	-0.25598	-0.50557	0.34	2	7	0	0	6	3	0	0
16	16b	16a	06/21/13	-0.77885	1.17638	1.37080	0.64	21	0	0	0	20	0	2	0
17	17a	17b	04/07/11	0.11774	0.21871	0.12426	0.61	13	3	3	1	10	2	0	2
17	17b	17a	04/19/11	-0.62606	-1.06974	1.38127	0.64	6	13	0	0	11	6	0	0
18	18a	18b	03/18/08	-0.81791	-0.80311	-0.50557	0.49	10	6	0	0	29	7	3	0
18	18b	18a	05/09/15	-0.56234	1.11740	-0.49183	0.72	10	2	0	0	37	4	0	0
19	19a	19b	04/02/02	-0.87906	-0.17886	-0.45397	0.70	3	8	0	0	5	4	0	0
19	19b	19a	06/01/16	0.06371	0.87658	-0.50157	0.55	7	0	4	4	0	3	0	8
20	20a	20b	03/18/01	-0.75412	0.39066	-0.34996	0.50	10	2	0	0	13	1	0	0
20	20b	20a	03/26/18	0.21382	0.01243	3.79300	0.39	14	0	2	0	18	1	1	0
